# Childhood trauma in bipolar disorder: experience of Arrazi hospital

**DOI:** 10.1192/j.eurpsy.2024.891

**Published:** 2024-08-27

**Authors:** H. Boukidi, H. Ballouk, M. Sabir, N. Ait bensaid, F. Elomari

**Affiliations:** Arrazi psychiatry hospital, Sale, Morocco

## Abstract

**Introduction:**

Bipolar disorder is a chronic, recurrent, and disabling condition that typically begins in late adolescence or early adulthood. It is characterized by alternating phases of depression, mania, or hypomania. Childhood traumas are more frequently found in adults with bipolar disorder, suggesting their contribution to its development. They are also associated with more severe and complex clinical forms and a less favorable prognosis.

**Objectives:**

Our objective is to assess the prevalence of childhood trauma rates in adults with bipolar disorder and to study the impact of childhood traumas on the clinical course of bipolar disorder, in comparison with a group of patients with bipolar disorder who did not experience trauma during their childhood.

**Methods:**

This is a descriptive cross-sectional study using a questionnaire comprising sociodemographic criteria and the Childhood Trauma Questionnaire Short Form (CTQ-SF) to evaluate the connection between physical and psychological traumas during childhood and bipolar disorder. The study also examines the types of these traumas and their impact on the course of bipolar disorder in these categories.

**Results:**

Data were collected from 54 patients with bipolar disorder at Ar-Razi Psychiatric University Hospital. Among this sample, 60% were female and 40% were male. The age of the participants in our study ranged from 18 to 54 years. According to the Childhood Trauma Scale, approximately one-third of patients with bipolar disorder had experienced childhood trauma. Moreover, most participants who had survived childhood trauma experienced more relapses than patients who had not experienced traumatic incidents during their childhood.

**Conclusions:**

Childhood traumas and bipolar disorder appear to have a significant causal association, both in the development of the disease and its course. The results of our study support evidence published in articles to better clarify the nature of this association. However, our study has several limitations, including a limited sample size and difficulties in long-term follow-up during the disease. Therefore, further studies exploring this subject are desirable for better management of this condition.

**Disclosure of Interest:**

None Declared

